# Shared Use of Physical Activity Facilities Among North Carolina Faith Communities, 2013

**DOI:** 10.5888/pcd14.160393

**Published:** 2017-02-02

**Authors:** Annie Hardison-Moody, Michael B. Edwards, Jason N. Bocarro, Anna Stein, Michael A. Kanters, Danielle Marie Sherman, Lori K. Rhew, Willona Marie Stallings, Sarah K. Bowen

**Affiliations:** 1 North Carolina State University, Raleigh, North Carolina; 2 North Carolina Division of Public Health, Raleigh, North Carolina; 3 Partners in Health and Wholeness, North Carolina Council of Churches, Raleigh, North Carolina

## Abstract

**Introduction:**

Shared use of recreational facilities is a promising strategy for increasing access to places for physical activity. Little is known about shared use in faith-based settings. This study examined shared use practices and barriers in faith communities in North Carolina.

**Methods:**

Faith communities in North Carolina (n = 234) completed an online survey (October–December 2013) designed to provide information about the extent and nature of shared use of recreational facilities. We used binary logistic regression to examine differences between congregations that shared use and those that did not share use.

**Results:**

Most of the faith communities (82.9%) that completed the survey indicated that they share their facilities with outside individuals and organizations. Formal agreements were more common when faith communities shared indoor spaces such as gymnasiums and classroom meeting spaces than when they shared outdoor spaces such as playgrounds or athletic fields. Faith communities in the wealthiest counties were more likely to share their spaces than were faith communities in poorer counties. Faith communities in counties with the best health rankings were more likely to share facilities than faith communities in counties that had lower health rankings. The most frequently cited reasons faith communities did not share their facilities were that they did not know how to initiate the process of sharing their facilities or that no outside groups had ever asked.

**Conclusion:**

Most faith communities shared their facilities for physical activity. Research is needed on the relationship between shared use and physical activity levels, including the effect of formalizing shared-use policies.

## Introduction

Increasing access to safe, affordable recreational facilities is one way to increase physical activity. The strategy is particularly important for increasing physical activity in low-income or racial/ethnic minority communities, where issues related to safe access to recreational facilities are well-documented (1,2). Shared use (working with organizations to open access to their facilities) is a promising strategy for increasing the number of safe and accessible spaces to be physically active (3,4). Although researchers have examined the prevalence of shared-use agreements in schools, little is known about implementation of and barriers to shared use of recreational facilities in faith-based settings (5).

Faith communities are effective partners for promoting healthful eating and physical activity among their members (6). Public health practitioners have harnessed this potential by implementing and evaluating obesity prevention programs that have included lay advisor models (7,8), targeted diabetes education (7), and community-based participatory research (9,10). Researchers have documented and evaluated how faith communities implement healthful eating or physical activity policies, provide spaces or time for physical activity, and promote access to healthful foods (5,8). 

Given the importance of ecological frameworks in the design and implementation of physical activity interventions, community and environmental supports for physical activity are increasingly promoted as a strategy to increase individual physical activity levels among community members (4,11). Shared-use policies and practices, mainly in school settings, are promoted as a way to increase physical activity levels, particularly in areas that lack spaces to be active (12–14). For example, in North Carolina, a high percentage of schools (88.9%) allowed shared use of their facilities, a percentage much greater than that found in previous studies (15,16). Shared use was disproportionately lower in schools in economically distressed counties and in schools with a greater proportion of black students (17). Schools reported several barriers to implementing shared-use policies, including concerns about liability and maintenance (16) and feelings among school administrators that the community lacked interest in shared use or that administrators did not know where to start this work (17).

Research on health promotion in faith communities is increasing (18). Formal (ie, written) or informal (ie, verbal) agreements and policies on shared use for health promotion purposes can regulate whether and how people or groups are allowed to use the facilities of faith communities. Some faith communities adopt formal facility-use policies that outline the conditions and costs associated with individuals and groups using their space for meetings (eg, for Alcoholics Anonymous, Boy Scouts) or events. Some faith communities permit unstructured use — sometimes called open use — of their recreational spaces by individuals and groups in the community. For example, many faith communities have playgrounds that are used by neighborhood children and families (19). A policy allowing unstructured use may be part of an intentional decision by the leaders of the faith community to make space available to the community for open recreational use and can lead to the adoption of a formal open-use policy. On the other hand, open use may be a customary practice of the faith community or neighborhood that has not been explicitly discussed or affirmed and is occurring with no policy — either formal or informal — in place.

The objectives of this study were to 1) create a baseline assessment of shared use of physical activity facilities among North Carolina faith communities, 2) determine barriers to shared use, and 3) determine priorities for future programs to support shared use of physical activity facilities in faith communities.

## Methods

We conducted a survey of faith-based organization in North Carolina in fall 2013. Three organizations collaborated to implement this assessment: North Carolina State University, Partners in Health and Wholeness, and the North Carolina Division of Public Health (DPH). North Carolina State University and DPH have a history of working together. In 2007, they collaborated to create the practice-tested faith-based intervention Faithful Families Eating Smart and Moving More (Faithful Families) (1). Partners in Health and Wholeness (PHW), sponsored by the North Carolina Council of Churches, certifies and supports congregations in their efforts to encourage healthful eating, physical activity, and tobacco cessation. As a part of its Community Transformation Grant (CTG) project, DPH worked with faith communities to promote shared use of their facilities (2). The institutional review board at North Carolina State University approved this research.

We adapted the survey used for this project from an existing survey of shared use that was administered in North Carolina public schools in 2013, which was based on an assessment developed by Spengler et al in 2011 (16,17). The survey asked faith communities whether their facilities (including meeting rooms, kitchens, gymnasiums, playgrounds, and athletic or open fields) were used by groups or individuals outside of the faith community’s membership. If facilities were available for outside individual or group use, the survey asked participants whether this use occurred through a formal policy or agreement (ie, a written contract), an informal policy or agreement (ie, verbal permission), or no policy or agreement (ie, permission to use the space had not been discussed). Faith communities that did not open their facilities to outside groups or individuals were asked a series of questions about the barriers to doing so, including liability, maintenance, not knowing where to start, and lack of space or interest. Participants were asked to what extent they agreed or disagreed (1 = strongly agree, 2 = agree; 3 = neither agree nor disagree; 4 = disagree; 5 = strongly disagree) with statements about common barriers to shared use, including not having been asked, not knowing where to start, concerns about liability, and concerns about maintenance cost. 

We administered the survey electronically, via Qualtrics (Qualtrics LLC), and permitted faith communities with limited access to the Internet to submit paper copies of the survey. Using program records, partners distributed the survey to faith communities that had participated in Faithful Families or the PHW program (262 faith communities). CTG coordinators throughout the state also distributed the survey to faith community contacts in their counties. Any faith community could participate, regardless of tradition or religious background. Emails were addressed generically but were tailored by local staff members (Faithful Families facilitators, PHW liaisons, or CTG coordinators) to be delivered to their local contacts in the faith community. The survey was completed by clergy, deacons, health committee members, and faith community members. Using survey distribution approaches developed from the tailored-design method (20), we sent a presurvey email to all potential participants. This email included information about the survey, a confidentiality statement, and an invitation to either complete the survey or share it with faith community partners. The survey distribution email included a link to the survey and a contact directory for regional and state partners who could assist with any survey questions. We sent 2 reminder emails to all participants after the initial survey email. The survey was open starting October 24, 2013, for 6 weeks, with an initial deadline of November 24, 2013. The deadline was later extended to December 13, 2013. We pilot-tested the questionnaire with a representative from 6 faith communities. The pilot testing demonstrated that the survey questions were clearly stated, and no questions were changed as a result.

Because the survey was distributed across several networks, we do not know how many faith communities received the survey. However, according to the Association of Religion Data Archives, North Carolina has more than 15,000 faith communities (21). Two hundred and thirty-four faith communities completed all of the shared-use questions on the survey and were included in the analysis.

The questionnaire comprised 25 questions related to shared use of facilities, including questions about the county where the faith community was located, congregation size, types of facilities that were shared, type of policy or agreement governing the use (informal, formal, or none), and perceived barriers to shared use.

Using census data and the county information reported by survey respondents, we measured the percentage of black residents living in the county where each participating faith community was located. We classified the percentage of black residents as low (≤10%), moderate (11%–30%), and high (≥31%) (22). We obtained health rankings data for each county from the 2013 University of Wisconsin Population Health Institute’s County Health Rankings, which ranked each North Carolina county “according to summaries of a variety of health measures,” with 1 being the healthiest and 100 being the least healthy (23). Because standardized county classification systems designating rural areas are lacking (24), we used data from the 2010 decennial census (25) on the percentage of the county’s population living in rural areas (ie, outside urban areas or urbanized clusters) to characterize the rurality of counties. We obtained economic data from the North Carolina Department of Commerce’s 2013 ranking of the state’s 100 counties based on economic well-being (26). The 40 most distressed counties were designated as Tier 1, the next 40 as Tier 2, and the 20 least distressed as Tier 3.

### Data analysis

We used descriptive statistics to describe faith community characteristics and type of shared use. We used binary logistic regression to examine differences between congregations that shared use and those that did not share use. The regressions focused on faith community size, county economic tier, county health ranking, percentage of county population living in rural areas, and percentage of black residents in the county as key explanatory variables. Initial unadjusted models were estimated without controlling for other variables, followed by models estimated that controlled for other variables. Statistical significance was established at *P* = .05.

## Results

Of the 234 faith communities that responded to the survey, 78 (34.4%) were small (<120 members), 75 (33.0%) were medium sized (120–299 members), and 74 (32.6%) were large (≥300 members) (Table 1). Forty-four (18.8%) faith communities in the sample were in the most economically distressed counties in North Carolina; 41.0% were in Tier 2 counties, and another 40.2% were in Tier 3 counties (Table 1). Survey respondents varied by type of position and included clergy, lay health leaders, deacons, PHW liaisons, and general members of the faith community. 

**Table 1:** Characteristics of the Sample of Faith Communities (N = 234) Participating in a Study of Share-Use Facilities for Physical Activity, North Carolina, 2013

Variable	No. (%^a^)
**Size of faith community, no. of members^b^ (median = 200)**	
Small (<120)	78 (34.4)
Medium (120–299)	75 (33.0)
Large (≥300)	74 (32.6)
**County economic tier^c^**	
Tier 1	44 (18.8)
Tier 2	96 (41.0)
Tier 3	94 (40.2)
**County health ranking^d^ (median = 49)**	
Low (64–100)	77 (32.9)
Middle (33–63)	79 (33.8)
High (1–32)	78 (33.3)
**Percentage of county population that is black^e^ (median = 20.7%)**	
Low (≤10)	67 (28.6)
Moderate (11–30)	89 (38.0)
High (≥31)	78 (33.3)
**Percentage of county that is rural^f^ (median = 42.7%)**	
Low (<20)	81 (34.6)
Moderate (21–49)	75 (32.1)
High (≥50)	78 (33.3)
**Share facilities**	
Yes	194 (82.9)
No	39 (16.7)
Did not answer question^g^	1 (0.4)

a Percentages may not sum to 100 because of rounding.

b Several faith communities reported their faith community size as a range and were thus not included in this analysis.

c Of the 100 counties in North Carolina, the 40 most distressed counties were designated as Tier 1, the next 40 as Tier 2, and the 20 least distressed as Tier 3. Data source: North Carolina Department of Commerce (26).

d Data source: University of Wisconsin Population Health Institute (23). Each county was ranked “according to summaries of a variety of health measures,” with 1 being the healthiest and 100 being the least healthy.

e Data source: US Census Bureau (22).

f Data source: US Census Bureau (25).

g Although 1 faith community did not respond to this question, it did answer questions related to use of facilities.

Of the 100 counties in North Carolina, 53 were represented in the survey (Figure). The distribution of the survey counties most likely reflects the interests and priorities of local PHW, CTG, and Faithful Families program staff. The largest number of faith communities that responded, by county, were from Wake County (27 respondents) and the second largest from Forsyth County (21 respondents).

**Figure:**
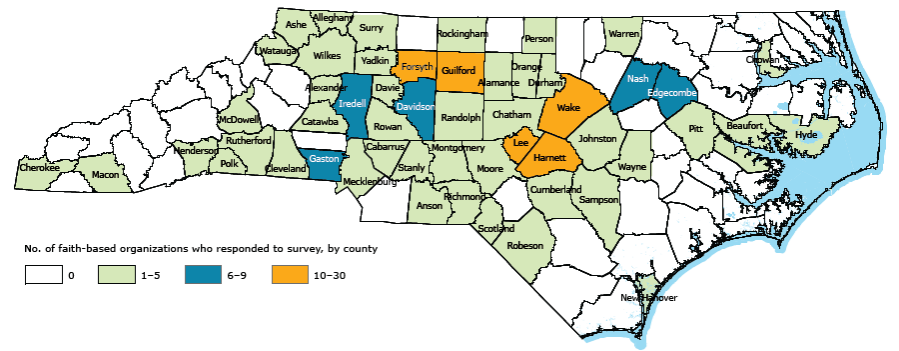
Counties represented by the faith communities that responded to the survey on sharing facilities for physical activity, North Carolina, 2013. **Table:** 

No. of Respondents	County
**0**	Avery, Bertie, Bladen, Brunswick, Buncombe, Burke, Caldwell, Camden, Carteret, Caswell, Clay, Columbus, Craven, Currituck, Dare, Duplin, Franklin, Gates, Graham, Granville, Greene, Halifax, Haywood, Hertford, Hoke, Jackson, Jones, Lenoir, Lincoln, Madison, Martin, Mitchell, Northampton, Onslow, Pamlico, Pasquotank, Pender, Perquimans, Stokes, Swain, Transylvania, Tyrrell, Union, Vance, Washington, Wilson, Yancey
**1–5**	Alamance, Alexander, Alleghany, Anson, Ashe, Beaufort, Cabarrus, Catawba, Chatham, Cherokee, Chowan, Cleveland, Cumberland, Davie, Durham, Henderson, Hyde, Johnston, Macon, McDowell, Mecklenburg, Montgomery, Moore, New Hanover, Orange, Person, Pitt, Polk, Randolph, Richmond, Robeson, Rockingham, Rowan, Rutherford, Sampson, Scotland, Stanly, Surry, Warren, Watauga, Wayne, Wilkes, Yadkin
**6–9**	Davidson, Edgecombe, Gaston, Iredell, Nash
**10–30**	Forsyth, Guilford, Harnett, Lee, Wake

Most respondents (82.9%) indicated that their faith communities had facilities that were shared with outside groups or individuals (Table 2). Of the 186 faith communities that had classrooms or meeting space, 167 (89.8%) shared them; 66.1% (39 of 59) shared gyms, 59.1% (68 of 115) shared playgrounds, and 55.1% (38 of 69) shared athletic/open fields (Table 2). Formal shared-use policies or agreements were more common for indoor facilities such as gyms (56.4%; 22 of 39) and classroom/meeting room spaces (51.5%; 86 of 167) than for outdoor spaces, whereas informal policies or agreements or no policy or agreement was more common for playgrounds (76.5%; 52 of 68), and athletic/open fields (63.2%; 24 of 38).

**Table 2:** Types of Most Frequently Shared Facilities and Types of Shared Use, Study of Share-Use Facilities for Physical Activity Among Faith Communities in North Carolina, 2013^a^

Type of Facility	Faith Communities That Have Type of Facility^b^ (N = 234)	Shared Facility^c^	Type of Agreement^d^			
			Formal	Informal	No Agreement	Did Not Indicate Type
Classroom/meeting space	186 (79.5)	167 (89.8)	86 (51.5)	76 (45.5)	3 (1.8)	2 (1.2)
Gymnasium	59 (25.2)	39 (66.1)	22 (56.4)	16 (41.0)	1 (2.6)	0
Playground	115 (49.1)	68 (59.1)	15 (22.1)	34 (50.0)	18 (26.5)	1 (1.5)
Athletic/open field	69 (29.5)	38 (55.1)	12 (31.6)	18 (47.4)	6 (15.8)	2 (5.3)
Other facility	194 (82.9)	188 (96.9)	NA^e^	NA^e^	NA^e^	NA^e^

a All values are number (percentage). Percentages may not add to 100 because of rounding.

b Percentage calculated according to number who responded to question (n = 234).

c Percentage calculated according to number of respondents that had the type of facility.

d Percentage calculated according to number of respondents that shared facility.

e NA, not applicable. Survey did not ask about type of policy or agreement for shared “other facilities.”

In unadjusted models, faith communities in the wealthiest (Tier 3) counties were significantly more likely (odds ratio [OR], 3.19; 95% confidence interval [CI], 1.10–9.25; *P* = .03) than faith communities in the poorest counties (Tier 1) to share facilities (Table 3). Similarly, faith communities in counties with the highest ranking in health outcomes were significantly more likely (OR, 3.32; 95% CI, 1.31–8.45; *P* = .01) to share facilities than faith communities in counties ranked lowest in health outcomes. In addition, faith communities in counties with a moderate percentage of rural residents were less likely (OR, 0.28; 95% CI, 0.11–0.71; *P* = .007) than faith communities with a high percentage of rural residents or a low percentage of rural residents to share facilities.

**Table 3:** Unadjusted Odds Ratios for Likelihood of Shared Facilities, by Faith Community and County Characteristics, Study of Shared-Use Facilities for Physical Activity in North Carolina, 2013

Characteristics	Odds Ratio (95% Confidence Interval)	*P* Value	Model *R* ^2^
**Size of faith community, no. of members**			
Small (<120)	1 [Reference]		0.03
Medium (120–299)	1.03 (0.46–2.27)	.94	
Large (≥300)	2.43 (0.94–6.31)	.07	
**County economic tier^a^**			
Tier 1	1 [Reference]		0.08
Tier 2	0.80 (0.34–1.92)	.62	
Tier 3	3.19 (1.10–9.25)	.03	
**County health ranking** b			
Low (64–100)	1 [Reference]		0.05
Middle (33–63)	1.64 (0.74–3.61)	.22	
High (1–32)	3.32 (1.31–8.45)	.01	
**Percentage of county population that is black^c^**			
Low (≤10)	1 [Reference]		0.04
Moderate (11–30)	0.20 (0.25–1.33)	.20	
High (≥31)	0.38 (0.58–4.22)	.38	
**Percentage of county that is rural^d^**			
Low (<20)	1 [Reference]		0.06
Moderate (21–49)	0.28 (0.11–0.71)	.007	
High (≥50)	0.47 (0.18–1.24)	.47	

a Of the 100 counties in North Carolina, the 40 most distressed counties were designated as Tier 1, the next 40 as Tier 2, and the 20 least distressed as Tier 3. Data source: North Carolina Department of Commerce (26).

b Data source: University of Wisconsin Population Health Institute (23). Each county was ranked “according to summaries of a variety of health measures,” with 1 being the healthiest and 100 being the least healthy.

c Data source: US Census Bureau (22).

d Data source: US Census Bureau (25).

Thirty-nine faith communities reported not sharing their facilities; of these, 29 answered the question on barriers. The barrier most frequently cited was not knowing how to start the shared-use process (mean score = 2.9; 7 of 29 [24.1%] reporting). The second most common reason given was not being asked by outside groups or individuals to share (mean score = 2.7; 7 of 29 [24.1%] reporting).

## Discussion

Our study yielded findings that can help shape future projects and practices related to shared use of physical activity facilities in faith communities. First, these data illustrated that faith communities are opening up their spaces for shared use. As the first study of shared use among faith communities, this study can encourage public health practitioners to expand or enhance partnerships with faith communities to encourage shared use. To aid in this work, and as a direct result of this research and a request by DPH, ChangeLab Solutions developed a guide to implementing shared-use practices in faith communities (19).

Second, our study showed that faith communities are sharing various types of spaces: classrooms, gymnasiums, playgrounds, and athletic fields. As a part of this study, we asked faith communities that shared facilities whether they were willing to share their information in a public database, which is now available online. Additional research is needed to understand how these spaces are used and whether use differs between faith communities that have formal agreements and faith communities that have informal agreements or no agreements.

Third, our study found that several factors were associated with differences in sharing facilities. Faith communities in counties with the greatest wealth and highest health rankings were more likely to share their facilities than faith communities in poorer counties and counties with lower health rankings. Additionally, faith communities with a moderate percentage of rural residents were less likely to share facilities than faith communities in counties with a high percentage or low percentage of rural residents. These findings correspond to findings from a study by Edwards et al (27), which notes that stereotypes about suburban communities as middle-class enclaves may be misguided. Instead, Edwards et al suggest that gentrification, population growth, and redevelopment have shifted poor residents to suburbs, “creating growing pockets of low-income residents concentrated in disadvantaged suburban communities” (27). Although our results are preliminary, they demonstrate the need for additional research on these demographic shifts and their effects on access to spaces for physical activity, particularly for low-income and racial/ethnic minority communities.

Fourth, our study found that faith communities did not cite liability concerns or maintenance costs as the primary reasons for not sharing their facilities. These finding are similar to those of another study in North Carolina that examined shared-use practices in schools (17). In our study, although faith communities did express concerns about maintenance and liability, the barriers most frequently cited were not knowing how to start the shared-use process and not being asked by outside groups or individuals to share their facilities. 

Our study identified faith communities as potentially untapped resources for shared use and increasing physical activity. From a social-ecological perspective, place-based physical activity interventions should consider the social, physical, and organizational environment to maximize usage and promotion of shared use of facilities for physical activity (18). A follow-up study examining the supporting practices and characteristics of faith community facilities with varying levels of shared use could provide a better understanding of the effectiveness of shared use and, more importantly, identify strategies for increased use of these facilities.

This study has several limitations. First, it was not designed to serve as a comprehensive assessment of the facilities of faith communities in North Carolina. Because the study was based on a convenience sample, the results may not be representative of all faith-based organizations in the state. Because we sent the survey to a wide network of practitioners and partners, we could not determine a baseline number of faith communities to whom the survey was distributed, and therefore cannot calculate a response rate. This research focused on how faith communities shared their facilities, not whether they used the facilities of another organization. Second, the organizations that completed the assessment might represent those most interested in the topic of shared use or in promoting physical activity. As a result, a higher number of faith-based organizations that allow community access to their facilities might be represented in our data. Our data suggest the need for additional, larger studies that examine shared use policies and practices in faith communities. Third, we did not include denomination or religious affiliation in the survey, which has led us to adapt the survey instrument to include that information for future use. Fourth, the logistic regression had limitations. Because of the small sample size, some variables had small numbers of outcome events, which led to wide confidence intervals for the odds ratios. Therefore, associations between variables should be interpreted with caution; a larger sample of faith communities is needed for more precise estimates.

Our findings suggest that faith communities are apt partners for increasing shared use in the community setting. Faith communities have facilities that can be used for various physical activities: indoor classrooms can be used for fitness classes; gymnasiums can be used for free play, games, or fitness classes; outdoor spaces can be used for organized games or free play; and large open spaces (including parking lots) can be opened up for biking, walking, and other activities. Faith community partnerships that promote shared use could be particularly important for communities that have persistent health disparities, including low-income and racial/ethnic minority communities, where access to spaces for physical activity may be limited.
